# Characterizing Curing Efficiency of EGCG-Encapsulated Halloysite Nanotube Modified Adhesives for Durable Dentin–Resin Interfaces

**DOI:** 10.3390/polym17010001

**Published:** 2024-12-24

**Authors:** Saleh Alhijji, Jeffrey A. Platt, Nassr Al-Maflehi, Abdulaziz Alhotan, Julfikar Haider, Marco C. Bottino, L. Jack Windsor

**Affiliations:** 1 Department of Dental Health, College of Applied Medical Sciences, King Saud University, P.O. Box 10219, Riyadh 12372, Saudi Arabia; smalhijji@ksu.edu.sa; 2 Department of Biomedical Sciences and Comprehensive Care, Indiana University School of Dentistry, Indianapolis, IN 46202, USA; jplatt2@iu.edu (J.A.P.);; 3 Periodontics and Community Dentistry Department, College of Dentistry, King Saud University, Riyadh 11545, Saudi Arabia; 4 Department of Engineering, Manchester Metropolitan University, Manchester M1 5GD, UK; 5 Department of Cariology, Restorative Sciences and Endodontics, School of Dentistry, University of Michigan, Ann Arbor, MI 48109, USA; 6 Department of Biomedical Engineering, College of Engineering, University of Michigan, Ann Arbor, MI 48109, USA

**Keywords:** dentin adhesive, MMP inhibitors, nanotube encapsulation, degree of conversion, dentin–resin interface

## Abstract

Matrix metalloproteinase (MMP)-induced collagen degradation at the resin-dentin interface remains a significant challenge for maintaining the longevity of dental restorations. This study investigated the effects of epigallocatechin-3-gallate (EGCG), a potent MMP inhibitor, on dental adhesive curing efficiency when encapsulated in halloysite nanotubes (HNTs). EGCG-loaded HNTs were incorporated into a commercial dental adhesive (Adper Scotchbond Multi-Purpose) at 7.5% and 15% *w/v* concentrations. To isolate the effects of each component, the study included three control groups: unmodified adhesive (negative control), adhesive containing only HNTs, and adhesive containing only EGCG (0.16% and 0.32%, equivalent to the EGCG content in EGCG–HNT groups). Degree of conversion (DC), polymerization conversion (PC), and Vickers micro-hardness (VHN) were assessed to evaluate curing efficiency. The addition of 7.5% EGCG-encapsulated HNTs maintained curing properties similar to the control, showing no significant differences in DC (80.97% vs. 81.15%), PC (86.59% vs. 85.81%), and VHN (23.55 vs. 24.12) (*p* > 0.05). In contrast, direct incorporation of EGCG at 0.32% significantly decreased DC (73.59%), PC (80.63%), and VHN (20.56) values compared to both control and EGCG–HNT groups (*p* < 0.05). Notably, HNT encapsulation mitigated these negative effects on polymerization, even at higher EGCG concentrations. These findings demonstrate that EGCG encapsulation in HNTs can maintain the curing efficiency of dental adhesives while potentially preserving the MMP-inhibitory benefits of EGCG.

## 1. Introduction

With the current trend of refraining from amalgam use due to mercury-related and esthetic concerns, resin composites are the most commonly used materials for direct restorations in dental clinics [1,2]. However, several studies have concluded that resins might be at greater risk for replacement and have a shorter lifespan than amalgam [3,4,5]. For instance, Mjör et al. [3] reported that resin composites required replacement more frequently due to secondary caries, while Soncini et al. [4] found significantly higher failure rates in composite restorations compared to amalgam, particularly in posterior teeth. The incidence of composite restoration failure is multifactorial and difficult to predict accurately [5]. Replacing restorations usually has undesirable side effects, such as increasing the cavity size, destroying the tooth, or requiring endodontic treatment or even extraction [6,7,8,9]. Secondary caries can develop at the dentin–resin junction due to the intermittent failure of the interfacial integration between the adhesive and the dentin organic structure in the hybrid layer [10,11]. Consequently, the degradation of the hybrid layer leaves a micro gap that allows cariogenic bacteria and their fermentation products to penetrate the interface, leading to the development of secondary caries [12]. Therefore, the integrity of the hybrid layer must be protected to ensure the longevity of the resin restoration [13].

Significant evidence available in the literature indicates that matrix metalloproteinases (MMPs) contribute to organic phase degradation at the resin–dentin interface by cleaving the collagen fibrils and subsequently creating a pathway for the development of secondary caries, which are associated with restoration failures [12,14,15,16], even in the absence of bacteria [17]. Furthermore, studies have shown that exposure to acidic etchant or resin components may activate MMPs in the dentin [18,19,20]. As a result, undesirable MMP activation can disrupt the interface and cause the restoration to debond. Therefore, it has been suggested that MMP inhibitors can be added to dental adhesives to prevent potential collagen cleavage at the hybrid layer and help extend the durability of the resin–dentin interface [21].

Epigallocatechin-3-gallate (EGCG), a catechin found in green tea, has shown promise as an MMP inhibitor and potential additive in dental adhesives [22,23,24]. Several studies have shown the effectiveness of EGCG in inhibiting a wide range of MMPs [25,26,27,28,29] and cysteine cathepsins [30], as well as inhibiting the growth and biofilm formation of cariogenic bacteria [24]. The incorporation of EGCG into a dental adhesive could represent a promising mechanism to protect the collagen fibrils in the hybrid layer after restoration placement. However, its direct incorporation into adhesives may affect curing efficiency and mechanical properties. To address this, the use of halloysite nanotubes (HNTs) as carriers for EGCG has been proposed [31].

The use of HNTs has been suggested to sustain the release of certain therapeutic and functional compounds [32,33]. HNTs are naturally occurring aluminosilicate clay minerals [Al_2_Si_2_O_5_(OH)_4_] that have a bilayer roll structure in a tubular form that offers the ability to hold different chemical molecules in the space between its layers for slow and sustained release (i.e., for hours, days, and months) [31]. The inner layer side of HNTs is positively charged, whereas the outermost layer is negatively charged. These charges differ due to the different inner and outer surfaces of Al(OH)_3_ and SiO_2_, respectively [34,35].

In our previous work, we demonstrated that incorporating EGCG and EGCG-encapsulated HNTs into dental adhesives effectively controlled EGCG release while maintaining the inhibitory effects of matrix metalloproteinase-9 (MMP-9) [36]. These findings suggested potential benefits for the longevity of dental restorations. However, the impact of such modifications on the curing efficiency of dental adhesives remains unexplored. The present study seeks to address this knowledge gap by evaluating the effects of EGCG and EGCG-encapsulated HNT addition on the curing efficiency of dental adhesives.

The degree of conversion (DC), polymerization conversion (PC), and Vickers micro-hardness (VHN) analyses were utilized to investigate the effects of incorporating HNT and EGCG into dental adhesive polymerization performance. These parameters are essential indicators of the adhesive’s curing efficiency and mechanical properties, which directly influence its clinical performance. By examining these aspects, we aim to provide an understanding of how EGCG and EGCG-encapsulated HNTs affect both the therapeutic potential and the physical properties of dental adhesives. This knowledge is crucial for developing improved dental materials that can extend the lifespan of dental restorations while maintaining optimal functional characteristics. Building upon findings from the author’s previous dissertation work [37], this study hypothesize that (1) the addition of EGCG-encapsulated HNTs will not significantly decrease the degree of conversion, polymerization conversion, or micro-hardness of the dental adhesive compared to the control; (2) direct addition of EGCG to the adhesive will result in decreased curing efficiency and mechanical properties; and (3) the addition of HNTs alone may have a positive effect on the curing efficiency and mechanical properties of the adhesive.

## 2. Materials and Methods

### 2.1. Preparation of Adhesive Groups

EGCG from Sigma–Aldrich (St. Louis, MO, USA) (95% pure extract) was dissolved in absolute ethanol to make a 43.6 mM (20 mg/mL) solution that was mixed with 2 g of pre-sieved (≤45 µm) HNTs (Dragonite HP, Applied Minerals Inc., New York, NY, USA). The EGCG–HNT mixture was vortexed for 5 min at 2000 rpm, stored overnight on a rack rotor at 6 rpm, and finally centrifuged at 3000 rpm for 30 min. Vacuum pressure (at 85 kPa) was applied twice before and after the final stirring and maintained for 30 min to remove any trapped air pockets. The supernatant was collected to accelerate the drying process and stored in a freezer for further analysis. Finally, the EGCG–HNT solution was dried in an incubator set at 37 °C.

The preparation of adhesive groups was performed as described in previous dissertation work [37]. Commercially available etch-and-rinse adhesive (Adper Scotchbond Multi-Purpose, 3M Oral Care, St. Paul, MN, USA) was mixed with 7.5% and 15% (*w*/*v*) of either EGCG-encapsulated HNT (EGCG–HNT) or HNT alone to produce four HNT-containing groups, as shown in Table 1. The HNT concentrations (7.5% and 15% *w*/*v*) were selected to maximize encapsulation capacity while maintaining optimal adhesive flow properties, as previous research demonstrated that HNT incorporation above 15% significantly increased viscosity and above 20% compromised physicochemical properties [38]. Two additional adhesive groups were prepared by directly adding EGCG with an amount approximated to match the EGCG loaded in the EGCG–HNT adhesive groups. The amount of EGCG in these HNT-free adhesive groups was estimated by analyzing the unloaded EGCG in the supernatant from the encapsulation process using UV/Visible spectrophotometry (Ultrospec 3100 pro, Amersham Biosciences, Temecula, CA, USA) [39].

The modified adhesive groups were mixed overnight in a dark room at an ambient temperature of 22 °C. PTFE rings 8 mm in diameter and 0.9 mm in thickness were used as molds to fabricate the disk-like samples. For easy release, the molds were placed on a glass plate covered with a disposable transparent film layer (Apollo, Write-On Transparency Film; ACCO Brands, Lake Zurich, IL, USA). The adhesives were injected into the mold using a special pipette designed for high-viscosity liquids (Pos-D^TM^, METTLER TOLEDO, Columbus, OH, USA). A second glass plate was then placed over the mold and gently pressed to ensure that no air bubbles were trapped. Each side was cured for 20 s using a blue-violet LED curing unit (Bluephase Style; Ivoclar-Vivadent, Amherst, NY, USA) at a suitable output intensity (≥850 mW/cm^2^), which was monitored using a visible curing light meter (Cure-Rite; LD Caulk/Dentsply, York, PA, USA). After curing, the specimens were stored in separate vials for 24 h at 37 °C in dark and under dry conditions before testing. Prior to testing, specimens were polished with 600–1200 grit SiC papers under water cooling and ultrasonically cleaned in distilled water.

### 2.2. Validate the Variations Between Samples

The specimens were inspected for imperfections after curing and weighed to ensure that they had a similar mass. An analytical scale with an accuracy of 0.1 mg (Mettler AE100, Mettler Instrument Corp., Hightstown, NJ, USA) was used to weigh the specimens. Additionally, the morphology and particle size of the HNTs were characterized using a field emission scanning electron microscope (FE-SEM) (ZEISS) to ensure consistent nanotube structure and dimensions across samples.

### 2.3. Degree of Conversion Procedure

Five specimens from each group were analyzed using Fourier-transform infrared (FTIR) spectroscopy (Jasco FT-IR 4100) equipped with a ZnSe attenuated total reflectance (ATR) crystal to determine the degree of conversion (DC%). The IR spectra were recorded in the range of 4000–400 cm^−1^ with a resolution of 4 cm^−1^ and 32 scans per spectrum. The DC% was calculated based on the change in the absorbance intensity of the aliphatic C=C peak at 1638 cm^−1^, relative to the aromatic C=C peak at 1608 cm^−1^ (internal standard). The aromatic C=C peak area was determined by averaging the absorbances at 1608 cm^−1^ (baseline: 1590–1623 cm^−1^) and 1582 cm^−1^ (baseline: 1569–1591 cm^−1^) [40]. The following equation was used to determine the degree of conversion (DC):

(1) $$DC\left(\%\right)=1-\left\{\frac{Cured(area\;under\;aliphatic\;C=C/area\;under\;aromatic\;C=C)}{Uncured(area\;under\;aliphatic\;C=C/area\;under\;aromatic\;C=C)}\times 100\right\}$$

### 2.4. Polymerization Conversion Procedure

The same specimens used for the degree of conversion analysis were immersed in a methanol solution to allow monomer leaching (unpolymerized residuals) before the polymerization conversion (PC%) was calculated [41]. The specimens were stored individually and protected from light using amber glass vials at room temperature for 35 days to elute any residual monomers. The methanol solution was replaced weekly to prevent saturation effects and keep monomer extraction relatively constant. Upon the completion of the extraction, the IR measurements were repeated for the aliphatic C=C peak at 1638 cm^−1^ with a baseline between 1650 and 1622 cm^−1^. The following equation was used to calculate the polymerization conversion (PC%) [41]:

(2) $$PC\left(\%\right)=1-\left\{\frac{∆area\left(before\;extraction-after\;extraction\right)under\;cured\;aliphatic\;C=C}{area\;under\;cured\;aliphatic\;C=C(before\;extraction)}\right\}\times \left\{100-DC\right\}$$

### 2.5. Vickers Microhardness Procedure

Vickers microhardness (VHN) was determined using a fresh set of specimens (n = 5 per group). Measurements were obtained using a micro-indentation hardness tester (LECO, LM248AT) equipped with a Vickers pyramidal diamond indenter. Prior to testing, specimens were polished with 600–1200 grit SiC papers under water cooling and ultrasonically cleaned in distilled water. A load of 100 g was applied for 15 s for each indentation. Three indentations were made on each flat side of the specimen, resulting in a total of six measurements per specimen. The indentations were spaced at least 1 mm apart and 1 mm from the specimen edges to avoid edge effects. The arithmetic mean of the six measurements was calculated to determine the VHN for each specimen.

### 2.6. Statistical Analysis

The data were analyzed using IBM SPSS Statistics (Version 26). Levene’s test was used to assess the homogeneity of variances for weight variation between specimens. One-way analysis of variance (ANOVA) was performed to determine statistically significant differences among the groups for weight variation, degree of conversion (DC), polymerization conversion (PC), and Vickers microhardness (VHN) measurements. When ANOVA indicated significant differences, post-hoc Tukey’s Honest Significant Difference (HSD) tests were conducted for multiple comparisons. The level of significance (α) was set at 0.05 for all statistical tests. Results are presented as mean ± standard deviation (SD). For specific comparisons between two groups, independent samples *t*-tests were used where appropriate.

## 3. Results

### 3.1. HNT Particle Characterization

**Figure 1:**
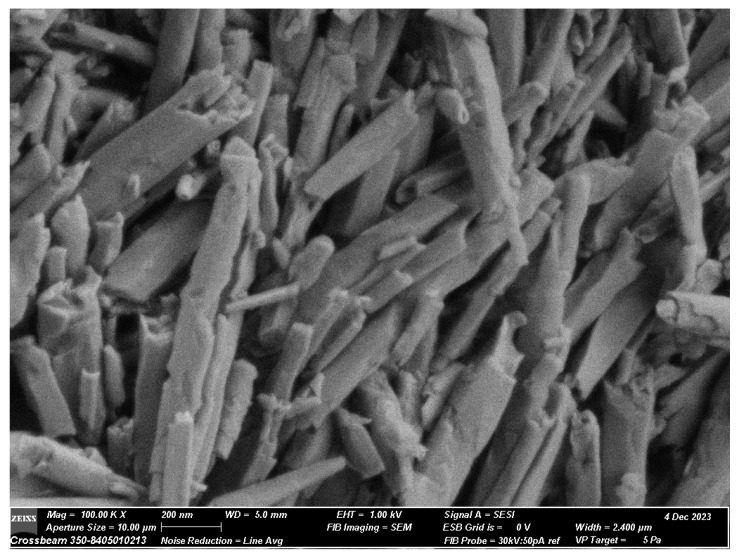
Representative SEM image of halloysite nanotubes (HNTs).

The morphology and particle size of the HNTs were characterized by FE-SEM, as presented in Figure 1. A long rod-like structure was observed in the HNT particles, with the hollow and open-ended structure clearly visible in the image. FE-SEM analysis revealed HNT dimensions ranging from 100 to 150 nm in diameter and 300–1500 nm in length. The uniform tubular morphology and consistent size distribution support their potential as drug carriers.

### 3.2. Weight Variation Between the Specimens

The mean values, standard deviations, and standard errors for the weight variation between the specimens are shown in Table 2. Levene’s test for homogeneity of variance revealed that the variances were homogeneous (*p* ≥ 0.05). One-way ANOVA revealed a nonsignificant difference in weight variability between the groups (*p* = 0.216).

### 3.3. Degree of Conversion Result

The minimum and maximum DC% observed ranged from 63% to 83%, with G7 (0.3% EGCG) exhibiting the lowest DC% and G5 (15% HNT) the highest (Table 3). The statistical analyses revealed that there was a statistically significant difference between at least two groups (F(6, 35) = [6.445], *p* < 0.001). Tukey’s HSD test for multiple comparisons revealed that the mean value of DC% was significantly different between the G7 (0.3% EGCG) group and all other groups except the G4 (0.15% EGCG) group at the *p* = 0.05 level. The difference between G7 (0.3% EGCG) and G1 (control group) was significant (*p* = 0.001, 95% C.I. = [−12.57, 2.54]).

### 3.4. Polymerization Conversion Result

**Figure 2:**
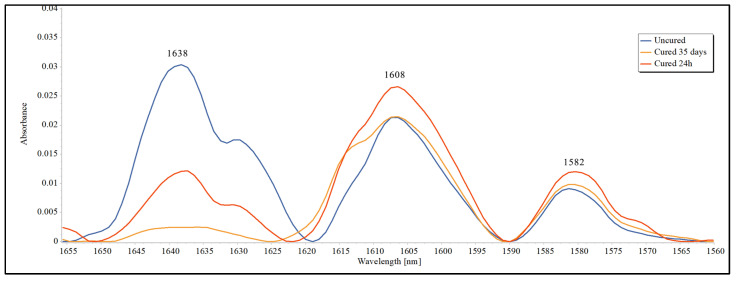
IR spectra (normalized) showing the peaks involved in the determination of DC% and PC% of the uncured adhesive, cured adhesive after 24 h, and cured adhesive after 35 days.

The area under the 1638 cm^−1^ peak was used to calculate the changes from the initial readings to 35 days (Figure 2). The statistical analysis revealed a statistically significant difference between at least two groups (F(6, 35) = [3.071], *p* = 0.016). Tukey’s HSD test for multiple comparisons revealed statistically significant differences between G7 and two groups: G3 (*p* = 0.032, 95% C.I. = [−11.58, −0.33]) and G5 (*p* = 0.007, 95% C.I. = [−12.66, −1.41]).

### 3.5. Vickers Microhardness Result

The Vickers microhardness results are summarized in Table 3. The statistical analysis revealed a statistically significant difference between at least two groups (F(6, 35) = [23.285], *p* < 0.001). Tukey’s HSD test for multiple comparisons revealed significant differences between G7 and G1 (*p* < 0.001, 95% C.I. = [−4.88, −2.22]) and between G4 and G1 (*p* = 0.01, 95% C.I. = [−2.93, −0.27]). However, there were no statistically significant differences between the HNT-containing groups (G2, G3, G5, and G6) and the control group (G1).

## 4. Discussion

This study investigated the effects of incorporating epigallocatechin-3-gallate (EGCG) and EGCG-encapsulated halloysite nanotubes (HNTs) on the curing efficiency of dental adhesives, building upon our previous work that demonstrated the potential of this system for controlled release of EGCG and MMP inhibition [36]. The degree of conversion (DC), polymerization conversion (PC), and Vickers microhardness (VHN) analyses were utilized to evaluate the impact on adhesive polymerization performance. Our results revealed a complex interplay between EGCG concentration, encapsulation status, and adhesive properties.

Our results directly addressed our three initial hypotheses. First, we confirmed that incorporating EGCG-encapsulated HNTs at 7.5% maintained curing properties comparable to the control adhesive, with no significant differences in DC (80.97% vs. 81.15%), PC (86.59% vs. 85.81%), and VHN (23.55 vs. 24.12) (*p* > 0.05). Second, direct EGCG addition significantly compromised curing efficiency, with 0.32% EGCG showing marked decreases in DC (73.59%), PC (80.63%), and VHN (20.56) compared to control (*p* < 0.05). Third, HNT incorporation alone showed a slight positive effect on curing properties, with 15% HNT achieving the highest DC (82.22%) and PC (87.67%) values among all groups.

Our findings regarding EGCG’s impact on adhesive properties align with several previous studies. Du et al. [42] observed a trend of decreasing DC with increasing EGCG concentration, though their changes were not statistically significant from the control group. In contrast, our study found statistically significant decreases in DC at higher EGCG concentrations (0.32%). This difference in statistical significance might be attributed to variations in EGCG purity (our study used 95% pure extract from Sigma-Aldrich) or concentration ranges tested. Khamverdi et al. [43] also observed adverse effects when incorporating EGCG into dental adhesives, although their study used different solvents. Conversely, Yu et al. [44] and Neri et al. [45] found no significant differences in bonding strength or DC with EGCG incorporation, but they used lower EGCG concentrations (0.01–1.00 mg/mL) compared to our study (1.6–3.2 mg/mL).

The positive effects we observed with HNT incorporation parallel findings by Bottino et al. [38], who reported that HNTs could enhance material properties up to certain concentrations. Similarly, our observation that 15% HNT showed optimal results but affected adhesive flow aligns with previous studies showing that HNT incorporation above 15% significantly increased viscosity and above 20% compromised physicochemical properties [31,32].

The PC calculation after 35 days revealed similar patterns to the DC results. The IR spectrum peak at 1638 cm^−1^ corresponds to an aliphatic carbon-to-carbon double bond, which should be at the highest value before curing. This can be attributed to the quantity of the resin monomer, as shown in Figure 1. Upon light curing, a growing chain reaction is initiated by converting the double bonds at the ends of the acrylic monomer to single bonds as the polymer network forms. In fact, the monomer conversion process is never complete, with the amount of unreacted residual monomer reported to be between 25% and 50% [46]. The reduction in the IR spectrum peak at 1638 cm^−1^ after 35 days is due to the washout of the monomer from the specimen. Therefore, the reduction in the area under this peak between the initial and 35-day readings was considered to indicate a higher degree of conversion and vice versa.

In the present study, EGCG was incorporated into the adhesive at final concentrations of 1.6 and 3.2 mg/mL (3.5 and 7.0 mM). This value may be greater than that reported in previous studies, which have typically incorporated EGCG into dental adhesives at concentrations ranging from 0.01 to 1.00 mg/mL (0.025 to 2.18 mM) [42,43,44,45,46,47]. Generally, no difference concerning bonding strength, flexural strength, or DC was observed between the EGCG-treated and control groups in these previous studies. However, one article reported adverse effects associated with a commercially available dental adhesive called the Filtek Silorane System [43]. The authors speculated that EGCG was not chemically compatible with the Filtek Silorane system. However, their study used water as a dilution solvent, which may interfere with the formula of some adhesives.

This study incorporated HNTs as drug carriers to elongate and sustain EGCG release. While the mechanism of molecular interaction between HNT and EGCG was not directly evaluated, the attraction likely involves oppositely charged ions, as supported by previous studies [34,35]. Based on our previous work [36], HNTs demonstrated the capacity to hold up to 21.35% (±4.6%) of EGCG during encapsulation. Future research should explore the release behavior of EGCG from HNTs under various physical and physiological conditions (i.e., temperature, pH, and pressure) to understand how its release can be altered in clinical use.

Photosensitizers in light-cured resin were added as the chemical reaction’s driving force to initiate the polymeric chain reaction. Determining the most effective amount of light (intensity) and its quality (i.e., wavelength) are crucial in the polymerization process and are dictated by the type of photoinitiator component in the resin [47]. It is expected that manufacturers will optimize the commercially available light-cured resins to achieve an efficient polymerization conversion. A commercially available dental adhesive was used in the current study rather than formulating an experimental adhesive to obtain a reliable baseline and reduce the number of variables that could require optimization and reassessment of the polymerization.

This study may have some methodological limitations that should be mentioned. While our standardized laboratory conditions allowed precise measurement of curing efficiency, clinical conditions may introduce additional variables, such as moisture and cavity configuration, that could affect polymerization. Our study focused on immediate post-cure properties, and our future research will examine long-term performance under simulated oral conditions. Additionally, while our findings demonstrate promising results with our selected commercial adhesive system, our future investigations with different adhesive formulations will help establish a broader applicability of this approach.

In conclusion, our hypotheses were largely supported by the results of this study. The use of EGCG-encapsulated HNTs appears to be a promising approach for incorporating MMP inhibitors into dental adhesives without significantly compromising their curing properties. However, the direct addition of EGCG, particularly at relatively high concentrations, negatively impacts curing efficiency and mechanical properties. These findings highlight the importance of the delivery method when incorporating bioactive compounds into dental materials.

Future studies should focus on evaluating the long-term stability of these modified adhesives, their bonding strength to dentin, and their performance under simulated clinical conditions. Additionally, investigating the release kinetics of EGCG from HNTs in the oral environment and its long-term MMP inhibitory effects would provide valuable insights for clinical applications.

### Clinical Significance

EGCG, which can inhibit MMPs and potentially bacterial growth, may enhance patient treatments by preventing the debonding of restoration caused by collagen degradation at the interface.

## 5. Conclusions

Within the limitations of this in vitro study, our findings reveal a complex interplay between EGCG, HNTs, and the adhesive system. HNT encapsulation proved advantageous in mitigating EGCG’s negative effects on adhesive polymerization, even at higher concentrations, demonstrating how the interactions between these components critically influence the system’s performance. Compared with the EGCG-only groups, the modified adhesives incorporated with 7.5% and 15% EGCG-encapsulated HNTs demonstrated efficient polymerization conversion. These findings represent a significant step toward developing dental adhesives that can provide both therapeutic benefits and optimal mechanical properties, potentially leading to more durable dental restorations. However, further clinical studies are needed to validate these laboratory findings and establish their long-term effectiveness in the oral environment.

## Figures and Tables

**Table 1:** The groups with different amounts of EGCG and HNT added to the adhesive.

ID	Group	HNT (mg/mL)	EGCG (mg/mL)
G1	Control adhesive	-	-
G2	7.5% HNT adhesive	75	-
G3	7.5% EGCG–HNT adhesive	75	1.6 *
G4	0.16% EGCG adhesive	-	1.6
G5	15% HNT adhesive	150	-
G6	15% EGCG–HNT adhesive	150	3.2 *
G7	0.32% EGCG adhesive	-	3.2

* Successfully loaded EGCG amount based on drug loading efficiency (21.35 ± 4.6%) as determined in our previous work [36].

**Table 2:** Results summary for the weight variation between the specimens involved in the study.

ID	Group	Weight (mg)	SD	Std. Err.	95% C.I. Lower Bound	95% C.I. Upper Bound
G1	Control adhesive	59.67	0.9	0.35	58.76	60.58
G2	7.5% HNT adhesive	60.10	1.6	0.66	58.41	61.79
G3	7.5% EGCG–HNT adhesive	59.90	1.2	0.48	58.68	61.12
G4	0.16% EGCG adhesive	59.47	1.1	0.44	58.34	60.60
G5	15% HNT adhesive	60.90	1.1	0.45	59.74	62.06
G6	15% EGCG–HNT adhesive	60.75	1.4	0.57	59.29	62.21
G7	0.32% EGCG adhesive	59.55	0.6	0.24	58.94	60.16

**Table 3:** Mean values and standard deviations of degree of conversion (DC), polymerization conversion (PC), and Vickers microhardness (VHN) across adhesive groups.

ID	Group	DC [%]	PC [%]	VHN
G1	Control adhesive	81.15 (0.62) ^a^	85.81 (1.24) ^a,b^	24.12 (0.33) ^a,b,c^
G2	7.5% HNT adhesive	80.54 (2.71) ^a^	84.63 (3.50) ^a,b^	24.32 (0.27) ^a,b^
G3	7.5% EGCG–HNT adhesive	80.97 (1.22) ^a^	86.59 (1.83) ^a^	23.55 (0.35) ^b,c,d^
G4	0.16% EGCG adhesive	78.29 (1.08) ^a,b^	84.55 (1.85) ^a,b^	22.51 (0.52) ^d^
G5	15% HNT adhesive	82.22 (1.33) ^a^	87.67 (1.63) ^a^	24.92 (0.21) ^a^
G6	15% EGCG–HNT adhesive	79.73 (1.07) ^a^	85.34 (1.77) ^a,b^	22.90 (0.71) ^c,d^
G7	0.32% EGCG adhesive	73.59 (6.38) ^b^	80.63 (6.45) ^b^	20.56 (1.65) ^e^

Different letters indicate significant differences at the 0.05 level.

## Data Availability

Data are contained within the article.
